# Emerging Roles of Long Non‐Coding RNAs in Cardiovascular Diseases

**DOI:** 10.1111/jcmm.70453

**Published:** 2025-03-03

**Authors:** Xiangyue Kong, Fengjuan Li, Yuan Wang

**Affiliations:** ^1^ Beijing Collaborative Innovation Centre for Cardiovascular Disorders, the Key Laboratory of Remodeling‐Related Cardiovascular Disease, Ministry of Education, Beijing Anzhen Hospital Capital Medical University Beijing China; ^2^ Beijing Institute of Heart, Lung, and Blood Vessel Diseases Beijing China

**Keywords:** biomarker, cardiovascular disease, Long non‐coding RNA, therapeutic strategies

## Abstract

Cardiovascular diseases (CVDs) are the leading cause of morbidity and mortality worldwide. Over the past decade, studies have demonstrated that circulating long non‐coding RNAs (lncRNAs)—recognised for their stability and ease of detection—serve as crucial regulators and potential biomarkers in multiple diseases. LncRNAs regulate key processes, including endothelial function, vascular remodelling, and myocardial hypertrophy, all of which influence CVD progression. Additionally, lncRNAs display cell‐, tissue‐, and disease‐specific expression patterns, making them ideal therapeutic targets or tools. This review presents a comprehensive overview of the current understanding of lncRNAs in CVDs, examining their mechanisms of action and recent research advances. It also addresses the use of lncRNAs as diagnostic and prognostic markers, as well as potential applications of RNA therapeutics in novel treatment strategies.

## 1 Introduction

Cardiovascular diseases (CVDs) are the leading cause of death worldwide [1]. An estimated 19.05 million deaths were attributed to CVD in 2020, which is an 18.71% increase from 2010 [2, 3]. Despite the implementation of various strategies to reduce the incidence of acute CVD and the associated mortality, the lasting effects of CVD continue to be a major contributor to chronic disease, disability, and death [4]. These effects have led to a growing economic burden on both developed and developing nations [5]. The pathogenesis of CVD is complex, and the absence of reliable, objective early diagnostic markers limits early detection and intervention. High‐sensitivity and high‐specificity cardiac marker assays are essential for early diagnosis of CVDs [6]. Thus, there is an urgent need to identify high‐risk individuals early and ensure they receive appropriate treatment to prevent premature death. A comprehensive understanding of the pathogenesis of CVD, coupled with the development of novel diagnostic and prognostic biomarkers and therapeutic strategies, is crucial for the reduction of CVD‐associated mortality.

In recent years, long non‐coding RNAs (lncRNAs) have been implicated in the development and progression of various CVDs [7, 8]. LncRNAs constitute a class of RNA molecules over 200 nucleotides long that do not encode proteins; studies have shown that the biogenesis of lncRNAs is closely linked to their subcellular localization and function [7]. LncRNAs can influence chromatin structure, membrane‐free nucleosome assembly and function, and cytoplasmic mRNA stability and translation; they can also modulate signalling pathways, depending on their interactions with DNA, RNA, and proteins. These functions impact gene expression across diverse biological and pathophysiological contexts [9]. LncRNAs are expressed in various cell types involved in CVDs and contribute to multiple biological processes active in these diseases, including endothelial dysfunction, smooth muscle cell homeostasis, lipid accumulation, and inflammation [7, 8, 10]. Additionally, circulating lncRNAs offer unique advantages as biomarkers in view of their stability, cost‐effectiveness, and ease of detection. Changes in blood lncRNA levels may be correlated with underlying disease mechanisms; thus, circulating lncRNAs are promising tools for early diagnosis and treatment of CVD [11, 12]. This review discusses the mechanisms of action of lncRNA and recent advances in research on CVDs, including their applications as diagnostic and prognostic markers and as targets for new therapeutic strategies.

## 2 Emergence and Biological Functions of lncRNAs

Most of the human genome encodes non‐coding RNAs, which are not translated into proteins [13, 14]. Since their discovery, non‐coding RNAs have demonstrated critical roles in regulating gene expression and organising the genome, shifting the view of RNA from a mere intermediary in protein synthesis to a functional molecule [15]. Initial studies in the late 1980s identified lncRNAs such as H19 and X inactivation‐specific transcripts (XIST) [16, 17]. The GENCODE project has estimated that there are 16,000 human lncRNAs, whereas the latest NONCODE database (v6.0) has annotated 96,411 human lncRNA genes, resulting in 173,112 lncRNA transcripts; this number is several‐fold higher than the estimated number of protein‐coding genes (approximately 20,000) [17, 18].

LncRNAs currently lack a standard classification but are commonly organised in the following ways. One classification method considers their transcriptional origin and spatial arrangement relative to adjacent genes, leading to categories such as sense, natural antisense, intronic, intergenic, bidirectional promoter, or enhancer [19]. Another approach categorises lncRNAs according to their molecular mechanisms, distinguishing between cis‐ and trans‐regulatory transcripts. Cis‐regulatory lncRNAs exert their effects at or near the transcription site, whereas trans‐regulatory lncRNAs act at distant chromosomal locations [19, 20]. LncRNAs can also interact with distant genomic regions because of the three‐dimensional structure of chromatin [21]. LncRNAs are also functionally classified based on their roles in the nucleus or cytoplasm. Nuclear lncRNAs participate in the recruitment of chromatin‐modifying enzymes, interactions with transcription factors, and regulation of splicing. In contrast, cytoplasmic lncRNAs control gene expression post‐transcriptionally by sequestering microRNAs or influencing the stability and translation of mRNA [20, 22].

Through interactions with DNA, RNA, and proteins, lncRNAs play essential roles in the pathogenesis of disease. Nuclear lncRNAs contribute to disease via the following mechanisms: regulation of downstream gene expression through chromatin remodelling and histone modifications; modulation of transcription by acting as enhancer RNAs; and influencing mRNA splicing by interfering with pre‐mRNA processing [21]. Cytoplasmic lncRNAs perform various roles, including the following: regulation and inhibition of specific transcription factors via decoy mechanisms; sequestration of microRNAs to control their stability and reduce their bioavailability via sponge mechanisms [23, 24]; control of gene expression by serving as molecular scaffolds that link proteins to form transcriptional or post‐transcriptional complexes; and binding proteins and participating in post‐translational modifications or acting as precursors to small RNA molecules [25].

## 3 Molecular Mechanisms of lncRNA Activity in CVDs

LncRNAs interact with various molecular components to modulate gene expression at the transcriptional, post‐transcriptional, and epigenetic levels, thereby influencing a range of cellular processes [26]. An understanding of the complex interactions between lncRNAs and various molecular components is essential when attempting to identify the roles of lncRNAs in CVDs and develop targeted interventions.

### 3.1 Transcriptional Regulation

In eukaryotic cells, gene transcription is controlled by protein complexes that bind to DNA promoters and RNA polymerase, modulating gene expression by activating or inhibiting transcription. These processes are crucial for various cellular processes [27]. Certain lncRNAs function as ligands, binding to transcription factors to form complexes. For instance, in hypertension‐associated vascular remodelling, PSR lncRNAs reportedly encode a novel protein known as arteridin, which interacts with transcription factor YBX1, promoting its translocation into the nucleus. The arteridin–YBX1–lncPSR complex formed regulates vascular remodelling via transcriptional regulation in vascular smooth muscle cells (VSMCs) [8]. Recent research has demonstrated that certain enhancers transcribe RNA that regulates genes in a specific orientation [7]. Enhancer‐associated lncRNAs are a newly identified class of lncRNAs that are transcribed from enhancer regions and typically shorter than 2 kb. These enhancers are characterised by specific histone modifications, including histone 3 lysine 27 acetylation and histone 3 lysine 4 monomethylation, as well as the recruitment of the histone acetyltransferase P300 [7, 28]. For example, the enhancer‐associated lncRNA PSMB8‐AS1 enhances PSMB9 expression by binding to the NONO and PSMB9 promoters. This interaction triggers ZEB1‐mediated upregulation of VCAM1 and ICAM1, contributing to vascular inflammation and atherosclerosis [7].

### 3.2 Post‐Transcriptional Regulation

LncRNAs also play a role in post‐transcriptional regulation of mRNA via alternative splicing, RNA editing, translation, and translocation [29]. These processes are essential for functional polymorphism in genes. Acting as competitive endogenous RNAs, lncRNAs can inhibit the degradation of target mRNA by competing with microRNAs and interfering with translational repression [30]. For example, MALAT1, a lncRNA, regulates VSMC proliferation, migration, and phenotypic transformation. It functions as a molecular sponge for miR‐145‐5p, increasing hexokinase 2 expression and influencing vascular remodelling in hypertension [31].

### 3.3 Epigenetic Modifications

LncRNAs may alter gene expression by recruiting chromatin remodelling complexes to specific loci, thereby altering DNA/RNA methylation status, chromosomal structure, and histone modifications. These changes impact gene expression, particularly in CVDs. The most common histone modifications in promoter regions are H3K4me3, H3K9me2, and H3K27me3; changes in these affect chromatin activity, which subsequently regulates gene expression and transcription [32]. For instance, the lncRNA ANRIL interacts with SUZ12 (a PRC2 component) and CBX7 (a repressor complex member), suppressing the expression of P15INK4b and P15INK4a genes. These epigenetic modifications may increase susceptibility to coronary heart disease by reducing the expression of P15INK4b and P15INK4a, which have anti‐atherosclerotic effects [33].

## 4 Clinical Implications of Identifying lncRNAs as Diagnostic or Prognostic Biomarkers of CVDs

LncRNAs not only function as key regulators but also hold promise as non‐invasive biomarkers [15]. Ideal biomarkers should be stable, highly specific, sensitive, simple to use, and cost‐effective [34]. lncRNAs can enter the circulation in the form of exosomes or microvesicles, and their stability in the bloodstream is attributed to their extensive secondary structures and the protection provided by exosomes during translocation [35, 36]. LncRNAs are usually detected using well‐established technologies such as RNA sequencing (RNA‐Seq) or quantitative polymerase chain reaction (PCR), which are widely used in laboratory and clinical settings. Unlike with traditional imaging examinations or invasive tissue biopsies, detection of lncRNAs requires only a small sample of blood, urine, or saliva, causing minimal discomfort to the patient and conserving medical resources [37]. Furthermore, the high specificity and sensitivity of lncRNAs can significantly reduce the risk of a missed diagnosis, thereby reducing unnecessary follow‐up examinations and their associated costs. Advances in portable detection technologies, such as microfluidic chips, are increasing the accessibility of lncRNA‐based diagnostics. These innovations are enabling more patients to receive timely and accurate diagnoses, ultimately improving treatment outcomes. Numerous studies have explored the diagnostic and prognostic potential of lncRNAs in CVDs. The lncRNAs identified as diagnostic or prognostic biomarkers in several CVDs are outlined in the following sections and summarised in Table 1.

**TABLE 1:** LncRNAs as biomarkers for the diagnosis and prognosis in CVDs.

Biomarkers									
Disease	LncRNA	Diagnostic biomarker	AUC	Sensitivity	Specificity	Regulation	Prognostic biomarkers	Species origin	Reference
Heart failure	NRF	Yes	0.975			Up	No	Human	[38]
	Heat2	Yes	0.705	58%	81.6%	Up	Yes	Human	[39]
	CASC7	Yes	0.85			Up	No	Human	[40]
	NEAT1 + miR‐129‐5p + BNP	Yes	0.998	98.6%	96.8%	Up	Yes	Human	[41]
	MHRT	No				Up	Yes	Human	[42]
	LUCAT1	Yes	0.933	90.43%	97.78%	Down	Yes	Human	[43]
	PVT1 + miR‐190a‐5p	Yes	0.984	92.4%	96.7%	Up	No	Mouse	[44]
	H19	Yes	0.631	53.6%	73%	Up	No	Human	[45]
	ZFAS1 + miR‐590‐3p + BNP	Yes	0.991	93.5%	97.5%	Up	Yes	Human	[12]
	LIPCAR	Yes	0.722	72.2%	62.3%	Up	Yes	Human	[45]
Myocardial infarction	TUG1	Yes	0.9038			Up	Yes	Human	[46]
	N1LR	Yes	0.873	72.3%	96%	Down	No	Human	[47]
	SNHG1	Yes	0.890	90.5%	80%	Up	No	Human	[47]
	LIPCAR	Yes	0.782	82%	75%	Early down, late up	Yes	Human	[48]
	ZFAS1	Yes	0.664			Down	Yes	Human	[49]
	CDR1AS	Yes	0.671			Up	Yes	Human	[49]
	CHAST	No				Early up, late down	Yes	Human	[50]
	UCA1	Yes	0.757			Early down, late up	No	Human	[51]
	TTTY15 + HULC	Yes	0.967			Up	No	Human	[52]
	XIST	Yes	0.886	94,8%	82.4%	Up	No	Human	[53]
Atherosclerosis	DANCR	Yes	0.947	78.1%	71%	Up	No	Human	[54]
	SENCR	Yes	0.769	76%	44%	Down	No	Human	[55]
	UC.98	Yes	0.811	69.6%	94.15	Up	No	Mouse	[56]
	MALAT1	No				Up	Yes	Mouse	[57]
	ATB	Yes	0.8787			Up		Human	[58]
	HIF1A‐AS	Yes	0.823			Up	No	Human	[59]
	XIST/SNHG5	No				Down	Yes	Human	[60]
Aortic aneurysm and Dissection	XIST	No				Up	Yes	Human	[61]
	LOXL1‐AS	Yes	0.95			Up	No	Human	[62]

### 4.1 Heart Failure

Heart failure (HF) is a potentially fatal condition that occurs in association with various CVDs. At present, clinical diagnosis of HF relies primarily on conventional biomarkers, such as brain natriuretic peptide (BNP) [63]. However, BNP levels can be significantly affected by other conditions, including hepatitis [64] and renal failure [65]. Moreover, while left ventricular ejection fraction is widely used to assess cardiac function in HF, it remains normal in some individuals with HF [66]. These limitations underscore the urgent need for the identification and development of more specific diagnostic and prognostic biomarkers for HF.

The potential of lnRNAs as biomarkers for HF has recently been explored. Xu et al. found that the expression of lncRNA‐CASC7 in plasma and peripheral blood mononuclear cells was significantly higher in patients with HF than in controls. The area under the curve (AUC) for lncRNA‐CASC7 was 0.85, indicating that it could effectively distinguish between patients with HF and controls [40]. Furthermore, Zhang et al. observed the upregulation of lncRNA NEAT1 in patients with HF. In their study, Kaplan–Meier analysis and multivariate Cox regression showed that a high NEAT1 level predicted a strong likelihood of overall survival in patients with HF (*p* < 0.01). Receiver‐operating characteristic (ROC) curve analysis indicated that serum NEAT1 had an AUC of 0.868 with a sensitivity of 62.9% and a specificity of 96.8%. Furthermore, when combined with BNP, based on an AUC of 0.98, NEAT1, miR‐129‐5p, and BNP together significantly enhanced the diagnostic accuracy [41]. In another study, elevated lncRNA ZFAS1 levels were observed in patients with HF. Combined assessment of ZFAS1 and the traditional biomarker BNP showed remarkable diagnostic efficacy, having an AUC of 0.991 with a sensitivity of 93.5% and a specificity of 97.5%. Furthermore, the Kaplan–Meier curves showed a correlation between a high ZFAS1 level and worse 24‐month survival, establishing ZFAS1 as an independent prognostic marker for HF [12]. These findings highlight the potential for the application of lncRNAs as diagnostic and prognostic biomarkers for HF, which may offer a more precise and comprehensive approach to managing this complex condition.

### 4.2 Myocardial Infarction

Diagnosis of myocardial infarction (MI) typically requires a combination of physical examination, electrocardiography, and detection of specific biomarkers [67]. In recent years, lncRNAs have emerged as promising diagnostic tools, demonstrating sensitivity and specificity superior to that of conventional MI biomarkers, such as the cardiac troponin isoforms and creatine kinase [68]. This enhanced performance facilitates prompt and accurate diagnosis, enabling early intervention and improving the prognosis. For example, Xie et al. compared plasma lncRNA levels between patients with acute myocardial infarction (AMI) and controls and found significant upregulation of the lncRNA level for TTTY15 and significant downregulation of that for HULC in patients with AMI. ROC curve analysis demonstrated that both TTTY15 and HULC outperformed the conventional AMI markers CKMB (creatine kinase–myocardial band) and troponin T, with AUCs of 0.915 for TTTY15 and 0.905 for HULC. Notably, a model that combined both these lncRNAs achieved an even higher AUC of 0.967 [52]. Whereas single biomarkers often have inherent limitations, the use of multiple biomarkers can provide insights into specific pathological mechanisms. This approach not only improves diagnostic accuracy but also aids in guiding targeted therapeutic strategies. For example, Zhu et al. found that lncRNA N1LR levels were significantly lower and lncRNA SNHG1 levels were significantly higher in their AMI group than in their control group. N1LR had an AUC of 0.873 with 96.0% specificity and 72.3% sensitivity for the diagnosis of AMI, whereas SNHG1 had an AUC of 0.890 with 80.0% specificity and 90.5% sensitivity. When combined, these lncRNAs had an AUC of 0.962, with 88.0% specificity and 94.6% sensitivity, highlighting their potential as diagnostic biomarkers for AMI [47].

In another study, the lncRNA TUG1 level was found to be significantly upregulated in patients with AMI compared to controls, with an AUC of 0.9038. Kaplan–Meier analysis further indicated that an elevated lncRNA TUG1 level was correlated with a poor prognosis in patients with AMI [69], suggesting its potential as both a diagnostic and prognostic marker. Wang et al. reported that lncRNA CHAST levels increased at 24 h post‐AMI but decreased significantly by day 3. CHAST also independently predicted cardiac contractile function in the early stages of AMI, indicating its value as a biomarker of left ventricular contractile function and remodelling during this critical phase [50].

In summary, there is a growing body of evidence that underscores the potential of lncRNAs in the diagnosis of MI and its prognosis. A deeper understanding of the molecular mechanisms underlying lncRNAs could significantly advance the diagnosis, treatment, and management of MI, ultimately improving patient care and outcomes.

### 4.3 Atherosclerosis

Atherosclerosis is a progressive condition that often develops silently, gradually impairing cardiovascular function and reducing the window for effective management after the onset of clinical manifestations. Traditional diagnostic methods, such as conventional angiography, are limited to the detection of significant stenosis but have been augmented by advanced anatomical and physiological techniques, including intravascular ultrasound and analysis of lipoprotein subclasses, to enable earlier diagnosis of atherosclerotic disease [70, 71]. Despite these advances, there remains an urgent need for more precise, non‐invasive diagnostic methods. Emerging research highlights the critical role of lncRNAs in atherosclerosis, positioning them as promising biomarkers for early diagnosis and prognosis.

Ye et al. observed significant downregulation of smooth muscle and endothelium‐enriched migration/differentiation‐associated lncRNA SENCR in the plasma of patients with coronary heart disease relative to controls. SENCR demonstrated an AUC of 0.769 with 76% sensitivity and 44% specificity. Spearman's rank correlation analysis revealed a negative correlation between the expression of SENCR and Gensini scores. This finding suggests that the SENCR expression level has an inverse relationship with the severity of coronary artery lesions [55]. Expression levels of lncRNA DANCR were elevated in serum samples from patients with atherosclerosis and positively correlated with low‐density lipoprotein cholesterol, homocysteine, and C‐reactive protein levels. ROC curve analysis demonstrated that DANCR effectively differentiated patients with atherosclerosis from controls, with an AUC of 0.947, sensitivity of 78.1%, and specificity of 71% [54]. These findings suggest that DANCR can serve as a diagnostic marker of atherosclerosis. In another study, Fan et al. used microarray analysis to examine the variability of lncRNA in a mouse model and identified a novel functional lncRNA, UC.98, which was associated with susceptibility to atherosclerotic plaque. This association was confirmed in peripheral blood samples from patients with atherosclerosis, in which UC.98 expression was positively correlated with the Gensini score, indicating a link between plaque instability and UC.98 expression patterns. With an AUC of 0.811, UC.98 demonstrated 69.6% sensitivity and 94.1% specificity for diagnosing complex lesions, indicating its potential as an early biomarker of atherosclerotic plaque [56].

A further study found that serum XIST and SNHG5 levels were lower and the miR‐155 level was higher in patients with atherosclerosis than in controls. Kaplan–Meier analysis revealed that the survival rate was lower in patients with low XIST/SNHG5 and high miR‐155 levels than in those with high XIST/SNHG5 and low miR‐155 levels. Specifically, survival was shorter in patients with atherosclerosis who had lower XIST and SNHG5 levels. Multivariate analyses further identified low XIST/SNHG5 expression, a high miR‐155 level, severe stenosis, and elevated triglycerides, low‐density lipoprotein cholesterol, and high‐sensitivity C‐reactive protein to be independent predictors of a poor prognosis in patients with atherosclerosis [60]. Overall, the integration of lncRNA biomarkers into diagnostic and prognostic frameworks represents a promising avenue for advancing the early detection and management of atherosclerosis.

### 4.4 Aortic Aneurysm and Dissection

Aortic aneurysm and dissection (AAD) presents significant diagnostic challenges in view of the overlap of its symptoms with those of other CVDs (e.g., AMI and pulmonary embolism) [72]. Current diagnostic methods are limited, and while some proteins have been explored as potential biomarkers for AAD, their specificity and sensitivity remain insufficient. Therefore, there is an urgent need to identify effective molecular biomarkers for the diagnosis of AAD [73]. Recent studies have highlighted the critical role of lncRNAs in the pathogenesis of AAD, positioning them as promising diagnostic and prognostic biomarkers. XIST has been implicated in aortic dissection. Zhang et al. found that the XIST expression level in aortic wall tissue was significantly higher in patients with aortic dissection than in those with normal aortic tissue. Kaplan–Meier analysis showed that survival was shorter in patients with higher XIST expression than in those with lower XIST expression, suggesting that upregulation of XIST is associated with a poor prognosis in patients with aortic dissection [61]. Similarly, Huang et al. found that the lncRNAs LOXL1‐AS and Giver were significantly elevated in patients with thoracic aortic aneurysm. ROC curve analysis demonstrated that LOXL1‐AS expression accurately distinguished patients with thoracic aortic aneurysm from controls, with an AUC of 0.95 [62], indicating high diagnostic accuracy. Overall, there is growing evidence that lncRNAs are important regulators in AAD. Early diagnosis could significantly improve outcomes of AAD.

## 5 Detection Techniques and Methods

The ability to detect lncRNAs is essential to understand their function and potential as biomarkers. LncRNAs have several advantages as biomarkers, in that they are highly stable and easily detectable; they can be accurately quantified using sensitive methods such as real‐time (RT)‐PCR, and changes in their levels may reflect disease mechanisms [74]. Common techniques used for lncRNA analysis include microarrays, RNA‐Seq, quantitative RT‐PCR, and NanoString technology.

The lncRNA microarray chip is designed to immobilise lncRNAs with known sequences on a glass or film chip using microprocessing technology. LncRNA expression profiling is performed on samples from patients and controls, and lncRNAs showing differences in expression are screened using bioinformatics methods. Candidates can be confirmed by quantitative RT‐PCR technology to determine their expression levels [75]. LncRNA sequencing reduces the abundance of rRNAs in a sample, allowing construction of libraries, sequencing, and analysis. This approach provides rapid and detailed information about lncRNAs associated with specific biological processes (e.g., development of disease) [76, 77]. RT‐PCR is widely used to detect lncRNA expression levels in a variety of sample types, including those containing low amounts of human RNA [78, 79]. NanoString technology allows simultaneous detection of multiple lncRNAs at the single‐molecule level, offering enzyme‐free detection and the ability to distinguish between cancerous and normal cells. This technology is valuable for live‐cell imaging and early clinical diagnosis [80].

### 5.1 Advantages and Disadvantages

Each of these methods has advantages and disadvantages, and the choice of technique often depends on specific research goals. The advantage of using microarrays is that they can be analysed in parallel with a high degree of automation, scale, and miniaturisation. However, they are expensive, generate a large and complex dataset, and require extensive bioinformatics to improve their accuracy [81, 82]. In contrast, RNA‐Seq provides a complete view of the transcriptome, revealing gene structure and expression levels in detail. It is efficient for identifying novel lncRNAs and overcomes the limitations of traditional microarray technology. However, RNA‐Seq is costly, requires high RNA purity, and involves cumbersome library preparation steps [76, 83]. Microarrays are often preferred over RNA‐Seq for lncRNA gene expression profiling in view of their efficiency and established protocols [75, 84]; however, they have lower specificity and a less dynamic range relative to quantitative RT‐PCR [80]. A combined approach that integrates RNA sequencing and microarrays has emerged for large‐scale clinical studies. Using this method, RNA‐Seq is performed at sufficient depth to identify all potentially relevant transcriptomic elements, after which custom‐designed arrays are used to analyse these elements in thousands of patient samples in a high‐throughput and reliable manner [84]. RT‐PCR remains the gold standard for validating lncRNA assays owing to its low cost and the relatively simple steps involved.

NanoString technology has several outstanding features. First, it is reproducible, accurate, and sensitive, based on barcode‐labelled fluorescent reporter probes with no need for enzymatic reactions or PCR amplification, and able to be performed independently of sample quality and without amplification errors. Second, it can quantify samples from various sources, including fresh or frozen tissue, cells, bacterial lysates, and formalin‐fixed, paraffin‐embedded tissue. Third, it requires a minimal sample volume, offers high throughput, and is user‐friendly, enabling accurate quantification of over 800 specific transcript sequences from as little as 100 ng of RNA [85]. However, its reliance on pre‐selected genes limits its applicability for the discovery of novel biomarkers.

In summary, the combination of RNA‐Seq, RT‐PCR, and NanoString technologies, along with advanced visualisation and computational tools, constitutes a comprehensive approach to the detection and analysis of lncRNAs. This multi‐faceted strategy is essential for elucidating the roles of lncRNAs in biological processes and diseases.

### 5.2 Potential Challenges in Detection of lncRNAs

Despite significant advances in the study of lncRNAs in cardiovascular disease, there are still many challenges to their clinical use as biomarkers, particularly the cost, time, reproducibility, and standardisation considerations involved in detecting lncRNAs compared to current standards such as protein‐based biomarkers.

The first challenge is the cost of detection of lncRNAs, which typically requires high‐throughput sequencing technologies such as RNA‐Seq and is expensive [86]. While quantitative RT‐PCR offers a more cost‐effective alternative, its reproducibility and accuracy are highly dependent on stringent experimental controls [79]. Various RNA isolation methods, normalisation methods, and the choice of quantitative RT‐PCR platform can affect the results, which increases the complexity and cost of the assay.

The second challenge is the time required for detection. LncRNA detection requires complex sample preparation and RNA extraction steps, which are not only time‐consuming but also require specialised technical operations. For example, preventing degradation of RNA during extraction is critical and increases the complexity and time cost of the operation [87]. Although high‐throughput sequencing technology can provide rich data, the detection cycle is long, usually requiring days or even weeks to complete the sequencing of samples and data analysis [88], making it less suitable for time‐sensitive applications. The third hurdle in lncRNA research is the lack of reproducibility of lncRNA data, which may be related to factors such as sample preparation and storage conditions, control of RNA degradation, and the presence of residual cells or hemolysis in body fluids. The choice of RNA isolation method and quantification platform can also have an impact on the results of an assay [89]. Future studies need to optimise the detection technology and improve the reproducibility and standardisation of data to facilitate broader use of lncRNAs in the diagnosis and monitoring of CVDs.

## 6 LncRNAs As Potential Therapeutic Targets in CVDs

A growing body of evidence indicates that lncRNAs serve as critical regulators in the pathophysiological processes that underlie various CVDs. With their unique regulatory functions, lncRNAs are increasingly recognised as novel therapeutic targets. One of the most intriguing aspects of lncRNAs is their tissue‐specific and spatiotemporal expression patterns. lncRNA expression varies between different types of tissue, and its expression in the same tissue or organ changes at different stages of growth [90]. This specificity makes lncRNAs particularly valuable for understanding disease mechanisms and developing targeted therapies. Studies comparing lncRNA expression across diverse sample types, ranging from individual cells to entire organs or organisms, have consistently demonstrated that lncRNA expression is more tissue‐ and context‐specific than that of protein‐coding genes [91].

**FIGURE 1:**
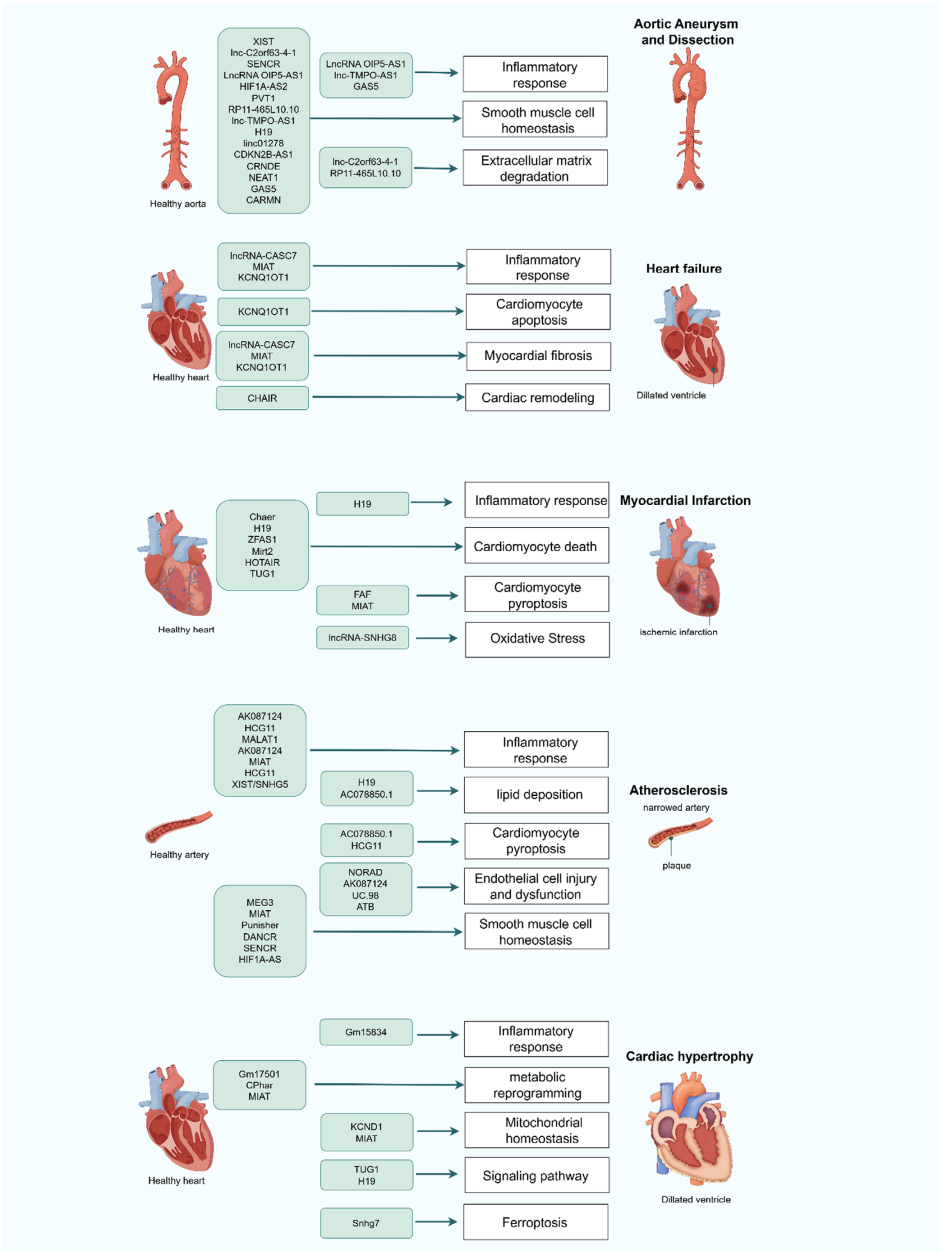
Mechanisms of lncRNA involvement in cardiovascular disease development.

In this review, we highlight the identification of lncRNAs that reveal novel pathological mechanisms and explore their significant influence on CVDs. We also provide a comprehensive summary of dysregulated lncRNAs implicated in the progression of CVDs (Table 2), offering insights into their potential roles in disease development and therapeutic strategies. These lncRNAs are involved in the development of cardiovascular disease by regulating various pathological processes, such as inflammatory responses, smooth muscle cell homeostasis, extracellular matrix degradation, and cardiac remodelling (Figure 1).

**TABLE 2:** LncRNAs as potential therapeutic targets in CVDs.

Potential therapeutic targets					
Disease	LncRNA	Function	Regulation	Species origin	Reference
Aortic aneurysm and Dissection	XIST (X‐inactive specific transcript)	Regulates vascular smooth muscle cell (VSMC) proliferation and apoptosis through the miR‐17/PTEN axis	Up	Human	[61]
	lnc‐C2orf63‐4‐1	Reduces angiotensin II (Ang II)‐induced signal transducer and activator of transcription 3 (STAT3) expression, controlling apoptosis and preventing synthetic phenotypic transformation in VSMCs	Down	Human	[92]
	SENCR (smooth muscle and endothelial cell‐enriched migration/differentiation‐associated lncRNA)	Downregulates miR‐206 expression, which prevents miR‐206 from inhibiting cardiac myosin, suppresses VMSC proliferation and migration, and maintains the contractile phenotype	Down	Human	[93]
	OIP5‐AS1	Exacerbates aorta intima, media, and adventitia injury in aortic dissection (AD) by upregulating TUB via sponging of miR‐143‐3p	Up	Human	[94]
	HIF1A‐AS2 (Hypoxia‐inducible factor 1 alpha‐antisense RNA 2)	Modulates SMC proliferation, migration, and phenotypic switching via miR‐33b/high mobility group AT‐hook2 (HMGA2) axis	Up	Human	[95]
	PVT1 (Plasmacytoma Variant Translocation 1)	Downregulation of PVT1 suppresses proliferation, migration, and phenotypic switching of platelet‐derived growth factor‐BB (PDGF‐BB)‐treated f human aortic smooth muscle cells (HASMCs) by targeting miR‐27b‐3p	Up	Human	[44]
	RP11‐465 L10.10	Induces VSMC phenotypic switching and matrix metallopeptidase 9 (MMP9) expression via the NF‐κB signalling pathway	Up	Human	[96]
	lnc‐TMPO‐AS1	Alkylation repair homologue protein 5 (ALKBH5) enhances AngII‐induced inflammatory response and apoptosis in HASMCs by regulating lnc‐TMPO‐AS1/EZH2/IRAK4 signalling through m6A modification	Down	Human	[97]
	H19	Involved in AD progression by mediating miR‐193b‐3p regulation of smooth muscle cell function	Up	Human	[98]
	linc01278	Regulates ACTG2 by sponging of miR‐500b‐5p to control phenotypic switching in VSMCs	Down	Human	[99]
	CDKN2B‐AS1	Facilitates STAT3 expression in VSMCs via competitive sponging of miR‐320d	Up	Human	[100]
	H19	Acts as a competitive sponge for let‐7a, increasing interleukin‐6 (IL‐6) expression during abdominal aortic aneurysm (AAA) development, promoting vascular inflammation and AAA formation	Up	Human	[101]
	CRNDE	Regulates VSMC proliferation and apoptosis through Smad3 and Bcl‐3	Down	Human	[102]
	PVT1	Exosomal PVT1 from macrophages regulates HMGB1 via ceRNA activity on miR‐186‐5p, promoting inflammatory pyroptosis	Up	Human	[103]
	NEAT1 (nuclear paraspeckle assembly transcript 1)	Positively regulates TULP3 via sponging of miR‐4688 in VSMCs; increased TULP3 expression inhibits VSMC proliferation and promotes apoptosis	Up	Human	[104]
	GAS5 (growth arrest‐specific 5)	Regulates the miR‐185‐5p/adenylate cyclase 7 (ADCY7) axis to modulate the AKT signalling pathway, promoting apoptosis while inhibiting proliferation and inflammatory responses in HASMCs	Up	Human	[105]
	CARMN	Protects against AAA formation and prevents VSMC phenotypic transformation by interacting with serum response factor (SRF), enhancing the effect of SRF on VSMC marker gene transcription	Down	Human	[106]
Heart failure	CHAIR (cardiomyocyte hypertrophic associated inhibitory RNA)	Reduces maladaptive cardiac remodelling by inhibiting DNMT3A binding to its target promoter	Down	Mouse	[107]
	NRF (necrosis‐related factor)	Circulating NRF levels show a positive correlation with troponin I (TnI) levels	Up	Human	[38]
	CASC7 (Cancer Susceptibility Candidate 7)	Acts as a competitive endogenous RNA for miR‐30c and indirectly regulates IL‐11 expression via miR‐30c	Up	Human	[40]
	Heat2	Elevated in immune cells of symptomatic HF patients, influencing proliferation, adhesion, invasion, and migration, likely through trans‐regulatory downstream mechanisms	Up	Human	[39]
	MIAT (myocardial infarction‐associated transcript)	Promotes inflammatory factor expression in myocardial fibrosis by activating the phosphoinositide 3‐kinase/protein kinase B (PI3K/AKt) pathway	Up	Human	[108]
	KCNQ1OT1	Targets Fused in sarcoma (FUS) proteins, downregulating their levels to promote apoptosis in cardiomyocytes of heart failure (HF) patients	Up	Mouse	[109]
Myocardial infarction	Chaer	Overexpression activates the AMP‐activated protein kinase (AMPK)/mTOR cascade, preventing cardiomyocyte death	Down	Mouse	[110]
	H19	Functions as a competitive endogenous RNA, regulating lysine (K)‐specific demethylase 3A (KDM3A) expression by binding miRNA‐22‐3p	Down	Mouse	[111]
	ZFAS1	Induces intracellular Ca^2+^ overload, triggering cardiomyocyte apoptosis	Up	Mouse	[112]
	FAF (FGF9‐associated factor)	Alleviates cardiomyocyte pyroptosis, enhances cell viability, and improves cardiac function by sponging of miR‐185‐5p, leading to increased P21 activated kinase 2 (PAK2) expression	Down	Mouse	[113]
	Mirt2 (myocardial infarction‐related transcription factors 2)	Sponges miR‐764 to promote 3‐phosphoinositide‐dependent kinase 1 (PDK1) expression, which inhibits cardiomyocyte apoptosis and mitigates myocardial infarction (MI)	Up	Mouse	[114]
	SNHG8 (small nucleolus RNA host gene 8)	Methyltransferase‐like 3 (METTL3) modifies m6A methylation to facilitate the binding of lncRNA‐SNHG8 and polyrimidine tract binding protein 1 (PTBP1), thus regulating ALAS2 expression, increasing oxidative stress, and promoting myocardial infarction	Up	Mouse	[115]
	NEAT1	Regulation of the miR‐378‐3p/Atg7 axis impacts cardiomyocyte proliferation and invasive metastasis	Up	Human	[116]
	HOTAIR (HOX antisense intergenic RNA)	Downregulates Bax and cleaved caspase‐3 protein levels while increasing Bcl‐2 expression	Down	Human	[117]
	MIAT (myocardial infarction‐associated transcript)	Promotes hypoxia‐induced cellular pyroptosis in H9C2 cells by inhibiting calcitonin gene‐related peptide transcription through binding to splicing factor 1	Up	Mouse	[118]
Atherosclerosis	H19	Serves as a ceRNA to upregulate Angiopoietin‐like 4 (ANGPTL4) expression by sponging of miR‐146a‐5p in THP‐1 cells	Up	Mouse	[119]
	AC078850.1	Regulates ITGB2 gene transcription by binding to hypoxia inducible factor‐1 (HIF‐1α). The lncRNA AC078850.1/HIF‐1α complex promotes NOD‐like receptor family pyrin domain containing 3 (NLRP3) inflammasome‐mediated pyroptosis and foam cell formation through a ROS‐dependent pathway in atherosclerosis	Up	Human	[120]
	NORAD (non‐coding RNA activated by DNA damage)	Inhibits vascular endothelial growth factor (VEGF) transcription in ox‐LDL‐treated human umbilical vein endothelial cells (HUVECs) by recruiting HDAC6 to VEGF promoters, enhancing H3K9 deacetylation	Up	Human	[121]
	MALAT1 (metastasis‐associated lung adenocarcinoma transcript 1)	Exhibits anti‐inflammatory properties, partly by interfering with miR‐503	Down	Mouse	[57]
	MEG3	Modulates the proliferation/apoptosis balance of vascular smooth muscle cells in atherosclerosis through the miR‐26a/Smad1 axis	Down	Human	[122]
	AK087124	Accelerates ox‐LDL‐induced endothelial cell injury by sponging miR‐224‐5p and increasing PTEN expression, leading to downregulated AKT rephosphorylation and upregulated apoptotic signalling	Up	Mouse	[123]
	MIAT (myocardial infarction‐associated transcript)	(1) Promotes SMC proliferation through the EGR1 (Early Growth Response 1)‐ELK1 (ETS Transcription Factor ELK1)‐ERK (Extracellular Signal‐Regulated Kinase) pathway, (2) activates pro‐inflammatory macrophages via NF‐κB signalling, and (3) mediates SMC phenotypic transdifferentiation to macrophage‐like cells by enhancing KLF4 transcriptional activity	Up	Human	[124]
	Punisher	Regulates apoptosis and mitochondrial homeostasis in vascular smooth muscle cells by targeting miR‐664a‐5p and OPA1	Down	Human	[125]
	HCG11 (HLA complex group 11)	Silencing of HCG11 inhibits cell pyroptosis and inflammation by targeting the miR‐224‐3p/Janus kinase 1 (JAK1) axis in ox‐LDL‐stimulated HUVECs	Up	Human	[126]
Cardiac hypertrophy	Gm15834	(1) Upregulated in cardiac hypertrophy, promoting hypertrophy through the miR‐30b‐3p/unc‐51‐like kinase 1 (ULK1) /autophagy pathway; (2) contributes to cardiac hypertrophy by binding to Sam68, activating NF‐κB, and inducing downstream inflammatory signalling pathways, which lead to hypertrophy	Up	Human	[127, 128]
	Snhg7	Transcriptionally regulates T‐box transcription factor 5 (Tbx5)/glutaminase 2 (GLS2)/ferroptosis in cardiomyocytes	Up	Mouse	[129]
	Gm17501	Modulates pathological hypertrophy and contributes to calcium handling pathways in cardiomyocytes	Early up，late down	Mouse	[130]
	Mhrt	Upregulates KLF4 expression through miR‐145a‐5p or inhibits KLF4 phosphorylation by ERK1/2, resulting in the suppression of myocardin expression and prevention of myocardial hypertrophy	Up	Mouse	[131]
	MIAT	Regulates CPT‐1a‐mediated cardiac hypertrophy via m6A RNA methylation of protein Ythdf2	Up	Mouse	[132]
	TUG1 (taurine up‐regulated gene 1)	Alleviates cardiac hypertrophy by targeting the miR‐34a/Dickkopf 1 (DKK1)/Wnt‐β‐catenin signalling pathway	Up	Mouse	[46]
	CPhar (cardiac physiological hypertrophy–associated regulator)	The RNA helicase DDX17 (DEAD‐Box Helicase 17) binds to CPhar, sequestering C/EBPβ (CCAAT/enhancer binding protein beta) away from the ATF7 (activating transcription factor 7) promoter, thus reducing ATF7's effects	Up	Mouse	[133]
	KCND1	Interacts with YBX1 and regulates cardiac mitochondrial function, playing a role in cardiac hypertrophy	Down	Mouse	[134]
	ZFAS1 (zinc finger antisense 1)	Promotes DNMT‐Notch1 interaction and facilitates DNA methylation‐mediated downregulation of Notch1, leading to increased cardiomyocyte apoptosis and reduced cardiac function	Up	Mouse	[135]
	H19	Interacts with polycomb repressive complex 2 (PRC2) to control nuclear factor of activated T cells (NFAT) signalling through epigenetic regulation of the Tescalcin locus	Down	Mouse	[136]
	CHRF (cardiac hypertrophy related factor)	Promotes cardiac hypertrophy by sequestering microRNA miR‐489, preventing suppression of its target mRNA, myeloid differentiation primary response gene 88 (Myd88)	Up	Mouse	[137]

### 6.1 Heart Failure

HF is characterised by structural and functional damage to the heart, impairing its ability to tolerate various hemodynamic and neurohormonal stresses [138]. LncRNAs play an important role in HF.

For example, Qian et al. identified cardiomyocyte hypertrophy‐associated inhibitory RNA (CHAIR), which is enriched in the heart. CHAIR is expressed at low levels in the embryonic heart, at high levels in the adult heart, and downregulated during HF. Recruitment of DNMT3A into the CHAIR promoter to repress its expression during heart failure ameliorates maladaptive cardiac remodelling. Restoration of CHAIR expression protects the heart from hypertrophy and failure [107]. In addition, Xu et al. found that plasma and peripheral blood mononuclear cells from HF patients showed high expression of the lncRNA CASC7, a competitive endogenous RNA for miR‐30c, which indirectly regulates the expression of IL‐11 through miR‐30. IL‐11 plays a strong pro‐fibrotic role in cardiac fibroblasts and increases ECM synthesis by cardiac fibroblasts, thereby affecting cardiac fibroblast migration and invasion. IL‐11 plays a strong pro‐fibrotic role in cardiac fibroblasts and can increase ECM synthesis in cardiac fibroblasts, thereby affecting cardiac fibroblast migration and invasion. The results of the present study provide new perspectives for targeted diagnosis and treatment of HF with lncRNA [40].

### 6.2 Myocardial Infarction

MI is characterised by acute necrosis of myocardial tissue resulting from prolonged, severe ischaemia and hypoxia. LncRNAs also play a key role in MI.

LncRNA small nucleolar RNA host gene 8 (lncRNA‐SNHG8) is a member of the lncRNA family. Through RNA sequencing, Zhuo et al. found that lncRNA‐SNHG8 regulates AMI [86]. In MI mouse models and human primary cardiomyocytes exposed to ischemia/reperfusion, Tang et al. observed abnormal overexpression of lncRNA‐SNHG8. The binding of lncRNA‐SNHG8 and PTBP1, which regulates ALAS2 expression, increases oxidative stress and promotes myocardial infarction. Silencing of lncRNA‐SNHG8 in cardiomyocytes, on the other hand, increased cardiomyocyte viability, decreased oxidative stress, and slowed myocardial infarction progression [115]. In addition, the lncRNA H19 was significantly downregulated after myocardial infarction. H19 plays a role as a ceRNA in the regulation of KDM3A expression through competitive binding to miRNA‐22‐3p. Enforced expression of H19 attenuates cardiomyocyte apoptosis and reduces inflammation, significantly reduces the size of myocardial infarction, improves long‐term cardiac function and attenuates cardiac fibrosis, whereas knockdown of H19 exacerbates symptoms of myocardial infarction [111].

Furthermore, compared to controls and untreated cells, ischemic cardiomyopathy/MI mouse cardiomyocytes showed significantly higher levels of lncRNA NEAT1. NEAT1 affects cardiomyocyte proliferation and invasive metastasis by regulating the expression of the miR‐378‐3p/Atg7 axis, suggesting that NEAT1 may be a potential therapeutic target for myocardial infarction. Inhibition of lncRNA NEAT1 reduced hypoxia‐induced apoptosis and enhanced cell migration and invasion, supporting its potential as a therapeutic target in MI [116].

### 6.3 Atherosclerosis

Atherosclerosis is a chronic vascular disease characterised by lipid accumulation in arterial walls, along with increased proliferation, migration, and apoptosis of VSMCs. It is a systemic, progressive disease resulting in the formation of fibrous atherosclerotic plaques that gradually encroach upon the vascular lumen, leading to vascular stenosis [139]. LncRNAs also play a regulatory role in atherosclerosis.

For example, patients with carotid plaques showed significantly higher levels of lncRNA AC078850.1 in their peripheral blood mononuclear cells relative to those without plaques; these levels were positively correlated with the severity of carotid atherosclerosis. The lncRNA AC078850.1 mediates cellular pyroptosis in atherosclerosis by increasing HIF‐1α‐mediated transcription of the ITGB2 gene. Knockdown of AC078850.1 inhibits macrophage pyroptosis and foam cell formation, thereby ameliorating AS [120]. Kai et al. found that the lncRNA NORAD (non‐coding RNA activated by DNA damage) inhibits VEGF gene transcription, increasing vascular endothelial cell injury and worsening atherosclerosis. Knockdown of NORAD reduced the development of atherosclerosis in mouse models [121].

### 6.4 Cardiac Hypertrophy

Cardiac hypertrophy can result from mechanical stress or neurohormonal stimulation. Sustained overload leads to reduced myocardial contractility and compliance over time, which are often accompanied by fibrosis, chronic inflammation, autophagy, and reactivation of fetal gene programs [140].

LncRNA Gm15834 is upregulated in cardiac hypertrophy and contributes to its pathogenesis through two distinct mechanisms. The first mechanism promotes cardiac hypertrophy via the miR‐30b‐3p/ULK1/autophagy pathway [127]. In the second mechanism, Gm15834 binds to Sam68, activating NF‐κB and inducing downstream inflammatory signalling pathways, thereby contributing to myocardial hypertrophy and the progression of cardiac hypertrophy [128]. In vivo inhibition of lncRNA Gm15834 reduces expression of Sam68, decreases inflammation, and mitigates cardiac hypertrophy [128]. Additionally, Yang et al. identified a novel lncRNA associated with hypertrophy—MIAT—which promotes myocardial hypertrophy during cardiac hypertrophy by modulating m6A RNA modification. Increased levels of MIAT have been linked to higher levels of cardiac hypertrophy [132].

### 6.5 Aortic Aneurysm and Dissection

Dysregulation of cellular and extracellular components in the aortic wall leads to progressive loss of smooth muscle cells, extracellular matrix destruction, and inflammation, resulting in AAD. Wang et al. found that lncRNA OIP5‐AS1 is highly expressed in the aortic tissue of patients with aortic dissection. This lncRNA upregulates TUB by acting as a sponge for miR‐143‐3p, inhibiting the proliferation and promoting apoptosis of human aortic endothelial cells and smooth muscle cells. It also induces the secretion of inflammatory factors that damage the aortic outer membrane, collectively exacerbating damage to the aorta's inner, middle, and outer membranes and contributing to the development of aortic dissection [94]. Moreover, Zhang et al. observed downregulation of lnc‐C2orf63–4‐1 during the progression of aortic dissection. This downregulation enhances signal transducer and activator of transcription 3 (STAT3) mRNA stability, activating downstream apoptotic signalling pathways, the matrix metalloproteinase family, and interstitial fibrosis factors. These molecular events lead to smooth muscle cell apoptosis, extracellular matrix degradation, and fibrosis, collectively contributing to vascular remodelling and exacerbation of aortic dissection [92]. Li et al. found that lncRNA CRNDE was downregulated in abdominal aortic aneurysm tissues and in angiotensin II‐stimulated VSMCs. Overexpression of CRNDE inhibited Smad3 protein ubiquitination by upregulating Bcl‐3, thus promoting VSMC proliferation and inhibiting apoptosis. This overexpression also attenuated abdominal aortic aneurysm by upregulating Smad3 expression [141]. The first lncRNA identified in the pathogenesis of thoracic aortic aneurysm was hypoxia‐inducible factor 1 (HIF1) alpha‐antisense RNA 1 (HIF1A‐AS1) [142]. Brahma‐related gene 1, which is elevated in thoracic aortic aneurysms, regulates HIF1A‐AS1 expression. Suppression of HIF1A‐AS1 decreases apoptosis and increases VSMC proliferation. Cardiac mesoderm enhancer‐associated non‐coding RNA (CARMN), a conserved lncRNA in smooth muscle cells, also plays a key role [143]. According to Liu et al., nuclear factor erythroid 2‐related factor 2 binds to the CARMN promoter, enhancing its transcription; CARMN reduces VSMC phenotypic changes and prevents aortic aneurysm formation [106]. These findings emphasise the crucial role of lncRNAs in AAD pathogenesis and highlight their potential as therapeutic targets.

The translation of animal model findings to humans remains challenging due to low lncRNA conservation across species [144]. The use of inducible pluripotent stem cells may help overcome some limitations of animal studies. LncRNA‐based therapies will benefit from identifying human‐specific lncRNAs, whether as human orthologs of lncRNAs [107] or as those expressed during embryonic stem cell differentiation into cardiomyocytes [145].

## 7 Targeted lncRNA‐Based Therapies

LncRNAs play critical roles in the initiation and progression of CVDs. Therapeutic modulation of lncRNA expression has been investigated in various preclinical in vivo models of cardiovascular conditions, including atherosclerosis, HF, and MI [146]. Studies have demonstrated that targeting lncRNAs for therapeutic purposes is feasible using antisense oligonucleotides, RNA interference (RNAi) technology, and CRISPR‐Cas9 [147]. Antisense oligonucleotides (ASOs) and RNAi have already been used in the clinical treatment of genetic diseases, and current research efforts are focused on improving the stability, specificity, and safety of targeted RNA agents. For example, chemical modifications, such as modification of mRNAs with pseudouridine, reduce immunogenicity and enhance efficacy. Furthermore, advances in delivery vectors, such as nanoparticle carriers, extracellular vesicles, and viral‐like vectors, have improved the potential for the use of these therapies in the treatment of CVDs. These advances have collectively paved the way for more precise and effective lncRNA‐based therapeutic strategies in cardiovascular medicine.

### 7.1 Antisense Oligonucleotides

ASOs are short deoxyribonucleotide analogs, typically 15–20 nucleotides in length, that are designed to bind complementary RNA sequences by Watson–Crick base pairing. This binding enables the reduction of target transcript expression, making ASOs powerful molecular tools for studying protein and RNA biology. They also represent a highly selective therapeutic strategy for diseases associated with dysregulated gene expression [148]. ASOs exert their effects via multiple mechanisms. An ASO essentially forms an RNA–DNA hybrid, leading to RNase H‐mediated cleavage of RNA. An ASO–RNA heteroduplex can also block splicing sites, causing inhibition of splicing or skipping of exons [149]. Targeting lncRNAs with ASOs holds promise as a therapeutic approach for treating CVDs. For example, an elevated blood lipoprotein(a) level is a risk factor for atherosclerotic CVD and aortic stenosis [150]. However, no therapeutic intervention is currently approved for lowering lipoprotein(a) levels. Pelacarsen, an ASO drug targeting mRNA for Apo(a) shows promise as the first effective lipid‐lowering agent for this purpose. In a Phase II clinical trial, lipoprotein(a) levels were reduced by 80% in patients who received pelacarsen, with 98% achieving levels of ≤ 50 mg/dL at a dose of 80 mg per month [151]. Moreover, ASOs are more effective than small interfering RNA (siRNA) or RNAi against nuclear lncRNA targets. This is because RNase H, the enzyme responsible for degradation of RNA, is predominantly localised in the nucleus [152]. ASOs are also known to modulate gene expression after transcription, but their effects on newly transcribed RNAs and potential adverse effects on target gene transcription remain unclear [148].

### 7.2 RNA Interference

RNAi has emerged as a powerful tool for genome‐wide high‐throughput screening, enabling researchers to study gene function across a range of cell types and organisms [153]. There are two types of RNAi, namely, arrayed siRNAs and lentiviral short hairpin RNA (shRNA) [154]. The use of siRNA technology to silence lncRNA COLCA1 in human coronary artery endothelial cells has demonstrated therapeutic potential. The use of siRNA technology reduced oxidative stress when these cells were stimulated by ox‐LDL in a model of CVD, significantly lowering the apoptosis rate, improving wound healing, and alleviating inflammation [155].

A combination of ASOs and siRNA can effectively inhibit lncRNAs with dual or ambiguous cellular localization [156]. A notable advance in this field is the “Smart Silencer,” a hybrid of ASO and siRNA containing three siRNAs and three ASOs that have been used in numerous in vivo and in vitro lncRNA knockdown studies [157, 158]. For example, an LncBAG6‐AS inhibitor containing three siRNA and three ASO sequences reduced the activity of manganese superoxide dismutase, glutathione reductase, and glutathione peroxidase in human umbilical vein endothelial cells [159]. It also decreased the mitochondrial membrane potential and alleviated oxidative stress in these cells. However, despite its widespread application, the efficacy of RNAi remains a matter of debate. Although numerous studies have demonstrated effective lncRNA knockdown [160, 161, 162], RNAi is less active in the nucleus, where many lncRNAs are predominantly located. This limitation renders RNAi less effective for targeting nuclear‐enriched lncRNAs [154]. Furthermore, the mechanism of action of RNAi is very imprecise. Introduced RNA molecules not only interact less specifically with off‐target transcript sequences but also interfere in an unpredictable manner with the cellular mechanisms that regulate gene expression [163]. Moreover, array siRNA screening has the disadvantage of requiring library synthesis, which is costly and will become obsolete with the development of lncRNA annotation.

To address the issue of off‐target effects, researchers have developed smaller antisense RNAs, such as locked nucleic acids, which have improved specificity. For example, small locked nucleic acids targeting the 5′ seed region of microRNAs can effectively antagonise and inhibit entire microRNA families. This approach has demonstrated therapeutic benefits, including improved cardiac parameters, in a mouse model of cardiac stress [164].

### 7.3 CRISPR‐Cas9

CRISPR‐Cas9 technology has revolutionised genetic engineering by enabling precise gene knockout through targeted DNA alterations, effectively eliminating gene expression with minimal off‐target effects. Integration of CRISPR‐Cas9 technology with high‐throughput functional genomics has enabled the identification of lncRNAs critical for targeted cardiovascular therapies, thereby facilitating customised and personalised treatments [156]. Furthermore, fusion proteins combining inactive Cas9 with a transcriptional activator domain could potentially upregulate the expression of lncRNA [165]. Moreover, the introduction of transcriptional stop signals can inhibit RNA polymerase activity, offering a versatile tool for gene regulation [166].

Despite these advances, CRISPR‐Cas9 continues to face challenges. A recent study showed that this technology was effective in only 38% of approximately 16,000 lncRNA loci tested [167]. Certain loci remain challenging to target with CRISPR‐Cas9 because of factors such as bidirectional, internal, or proximal promoters as well as off‐target effects on nearby genes [167, 168]. To address these limitations, researchers have developed a CRISPR‐Cas13‐based RNA‐targeted CRISPR screening method that allows functional screening at the transcriptome level in a transcript‐specific and strand‐specific system, overcoming the limitations of traditional methods [169].

The CRISPR/Cas (an acronym for “clustered regularly interspaced short palindromic repeats [CRISPR] and their associated proteins [Cas]) is the natural immune system” of microorganisms [170]. As a molecular diagnostic tool, CRISPR/Cas relies on its ability to specifically recognise and cleave nucleic acid sequences [171]. Three Cas proteins (Cas12, Cas13, and Cas14) with trans‐cutting activity are activated to cleave non‐target nucleic acids, which further amplifies the detection signal and improves detection sensitivity [172]. Meanwhile, the specific recognition of target sequences by guide RNA further improves the detection specificity. Therefore, the CRISPR/Cas diagnostic system is characterised by high sensitivity and high specificity [173].

CRISPR diagnostics is a next‐generation molecular diagnostic technology that uses RNA to detect biomarkers in biological fluids such as blood or urine. The process involves sample pre‐processing (including pre‐amplification), target identification, signal release, and detection [172]. In 2022, researchers developed a CRISPR assay called “CrisprZyme,” which can be performed at room temperature and can speed up the diagnosis of non‐communicable diseases such as heart disease. Unlike traditional CRISPR diagnostics, CrisprZyme uses colorimetric analysis to determine the number of biomarkers and was found to enable quantitative detection of the standard synthetic lncRNA LIPCAR (lnc‐LIPCAR) in the picomolar range. The researchers then examined the expression of lnc‐LIPCAR in the blood of 59 patients with acute chest pain and found that it was upregulated in patients with a high‐sensitivity troponin T level above 6 pg/mL [174]. This suggests that the CrisprZyme assay is able to identify a broad range of patients with HF, demonstrating the potential application of CrisprZyme in the diagnosis of severe myocardial injury.

### 7.4 Delivery of RNA Therapy

LncRNAs show potential as novel therapeutics; however, their physicochemical properties (e.g., hydrophilicity, negative charge, and instability) and biopharmaceutical limitations (e.g., low serum stability, rapid renal clearance, interactions with extracellular proteins, and limited cellular uptake) reduce their bioactivity and hinder effective delivery [175]. To overcome these barriers, advanced delivery systems such as exosomes, nanoparticles, and liposomes are being explored in the hope of achieving targeted delivery and sustained therapeutic efficacy [176]. Ideal delivery systems should be biocompatible, stable, cell‐permeable, and non‐immunogenic.

Delivery vectors for lncRNAs are broadly categorised into viral and non‐viral systems. Among the viral vectors, lentiviruses are widely used owing to their high transfection efficiency and ability to achieve prolonged gene expression. They are frequently used in experimental settings to modulate lncRNA levels, either by overexpressing specific lncRNAs or delivering siRNAs to knock down target lncRNAs [177, 178]. Adenoviral vectors are another option for modulation of lncRNAs, offering high transduction efficiency and transient expression [179]. For example, the delivery of lncRNA H19 via adeno‐associated virus 9 has been shown to reverse transverse aortic constriction‐induced pathological cardiac hypertrophy [180].

In preclinical studies, non‐viral delivery systems, particularly liposomes and nanoliposomes, are becoming increasingly popular lncRNA carriers [136]. Liposome encapsulation improves drug stability, enhances liver targeting, and reduces toxicity and immunogenicity by allowing drugs to bypass kidney filtration and undergo gradual absorption by liver cells [181]. Furthermore, extracellular vesicles, such as exosomes, have shown promise in regulating pathogenic lncRNA expression in diseases where lncRNAs play a dominant role [182].

Despite the immense potential of lncRNA‐based therapies, the field is still in its early stages, with several technical and ethical challenges to address. Furthermore, the long‐term safety and efficacy of lncRNA‐targeted therapies remain unclear, necessitating the establishment of robust long‐term monitoring mechanisms to evaluate their impact in the long term. Addressing these challenges will be critical for the successful translation of lncRNA therapeutics into clinical applications, particularly in the prevention and treatment of CVDs.

## 8 Conclusions and Perspectives

While significant progress has been made in characterising the functional diversity of lncRNAs, our current understanding represents only a fraction of their regulatory potential. Many aspects of lncRNA biology remain poorly understood, particularly the relationships between lncRNA sequences, their structural features, and their functions. This complexity is compounded by the non‐coding nature of lncRNAs and their low sequence conservation, which pose challenges for functional annotation and mechanistic studies. In recent years, studies of lncRNAs in CVDs have revealed their diverse roles in pathological processes underlying conditions such as MI, HF, atherosclerosis, and AAD. These lncRNAs exert their influence via various mechanisms, including acting as molecular decoys, regulating transcription and translation, influencing protein stability, and participating in chromatin remodelling. Despite their significant impact on the onset and progression of CVDs, the precise mechanisms by which lncRNAs contribute to these pathologies remain largely elusive. This knowledge gap underscores the importance of further mechanistic research, which could provide critical insights into the development of novel diagnostic, prognostic, and therapeutic strategies for CVDs.

A key unresolved question in the field is how dynamic changes in lncRNA expression reflect disease progression. Understanding these changes could offer valuable biomarkers for tracking disease severity and monitoring responses to treatment. Future research is expected to elucidate the specific mechanisms of lncRNAs, potentially paving the way for the development of targeted therapies and drug candidates. However, the majority of current investigations remain in the early stages of basic research, with limited translation into clinical applications.

Future studies should prioritise the following areas:

1. Mechanistic insights, which will allow us to delve deeper into the molecular pathways through which lncRNAs influence disease processes, focus on their interactions with proteins, DNA, and other RNAs.
2. Development of biomarkers, allowing exploration of the ability of lncRNA expression patterns to serve as dynamic biomarkers of disease progression and therapeutic efficacy.
3. Therapeutic targeting, by investigating the feasibility of targeting lncRNAs for therapeutic interventions while addressing potential off‐target effects and safety concerns.
4. Standardisation and reproducibility, by establishment of standardised methodologies for isolation, quantification, and functional analysis of lncRNAs, to ensure robust and reproducible results across studies.
5. Clinical translation involves accelerating the translation of lncRNA research into clinical applications by fostering collaborations between basic scientists and clinicians and by conducting well‐designed clinical trials.

By addressing these challenges, the full potential of lncRNAs as diagnostic tools, prognostic markers, and therapeutic targets can be unlocked, ultimately improving outcomes for patients with CVDs and other complex diseases.

## Author Contributions

**Xiangyue Kong:** writing – original draft (equal). **Fengjuan Li:** supervision (equal), writing – review and editing (equal). **Yuan Wang:** conceptualization (equal), resources (equal).

## Conflicts of Interest

The authors declare no conflicts of interest.

## Data Availability

Data sharing not applicable to this article as no datasets were generated or analysed during the current study.
